# Early impairment of intracranial conduction time predicts mortality in deeply sedated critically ill patients: a prospective observational pilot study

**DOI:** 10.1186/s13613-017-0290-5

**Published:** 2017-06-12

**Authors:** Eric Azabou, Benjamin Rohaut, Nicholas Heming, Eric Magalhaes, Régine Morizot-Koutlidis, Stanislas Kandelman, Jeremy Allary, Guy Moneger, Andrea Polito, Virginie Maxime, Djillali Annane, Frederic Lofaso, Fabrice Chrétien, Jean Mantz, Raphael Porcher, Tarek Sharshar

**Affiliations:** 1 Department of Physiology – Assistance Publique Hôpitaux de Paris, Raymond-Poincaré Hospital, INSERM U 1179, University of Versailles Saint-Quentin en Yvelines, Garches, France; 2 General Intensive Care Unit – Assistance Publique Hôpitaux de Paris, Raymond-Poincaré Hospital, INSERM U 1173, University of Versailles Saint-Quentin en Yvelines, Garches, France; 3 Department of Neurology, Intensive Care Unit, Groupe Hospitalier Pitié-Salpêtrière, AP-HP, Paris, France; 4 UPMC Univ. Paris 06, Faculté de Médecine Pitié-Salpêtrière, Sorbonne Universités, Paris, France; 5 Department of Anesthesiology and Intensive Care Medicine – Beaujon Hospital, University of Denis Diderot, Clichy, France; 6 Laboratory of Human Histopathology and Animal Models, Institut Pasteur, 28, rue du Dr Roux, 75015 Paris, France; 7 Department of Anesthesiology and Intensive Care Medicine – European Hospital Georges Pompidou, Paris Descartes University, Paris, France; 8 Center for Clinical Epidemiology - Assistance Publique Hôpitaux de Paris, Hotel Dieu Hospital, INSERM U1153, University Paris Descartes, Paris, France; 9 General Intensive Care Medicine, Raymond Poincaré Hospital (AP-HP), University of Versailles Saint-Quentin en Yvelines, 104, Boulevard Raymond Poincaré, 92380 Garches, France

**Keywords:** Prognostic, Outcome, Deep sedation, Evoked potentials, ICU, Brain dysfunction, Neurophysiological assessment, Delirium, Altered mental status

## Abstract

**Background:**

Somatosensory (SSEP) and brainstem auditory (BAEP) evoked potentials are neurophysiological tools which, respectively, explore the intracranial conduction time (ICCT) and the intrapontine conduction time (IPCT). The prognostic values of prolonged cerebral conduction times in deeply sedated patients have never been assessed. Sedated patients are at risk of developing new neurological complications, undetected. In this prospective observational bi-center pilot study, we investigated whether early impairment of SSEP’s ICCT and/or BAEP’s IPCT could predict in-ICU mortality or altered mental status (AMS), in deeply sedated critically ill patients.

**Methods:**

SSEP by stimulation of the median nerve and BAEP were assessed in critically ill patients receiving deep sedation on day 3 following ICU admission. Deep sedation was defined by a Richmond Assessment sedation Scale (RASS) <−3. Mean left- and right-side ICCT and IPCT were measured for each patient. Primary and secondary outcomes were, respectively, in-ICU mortality and AMS defined as the occurrence of delirium and/or delayed awakening after discontinuation of sedation.

**Results:**

Eighty-six patients were studied of which 49 (57%) were non-brain-injured and 37 (43%) were brain-injured. Impaired ICCT was a predictor of in-ICU mortality after adjustment on the global Sequential Organ Failure Assessment score (SOFA) [OR (95% CI) = 2.69 (1.05–6.85); *p* = 0.039] and on the non-neurological SOFA components [2.67 (1.05–6.81); *p* = 0.040]. IPCT was more frequently delayed in the subgroup of patients who developed post-sedation AMS (24%) compared those without AMS (0%). However, this difference did not reach statistical significance (*p* = 0.053). Impairment rates of ICCT and IPCT were not found to be significantly different between non-brain- and brain-injured subgroups of patients.

**Conclusion:**

In critically ill patients receiving deep sedation, early ICCT impairment was associated with mortality. Somatosensory and brainstem auditory evoked potentials may be useful early warning indicators of brain dysfunction as well as prognostic markers in deeply sedated critically ill patients.

## Background

While current guidelines advocate discontinuing sedation as soon as possible [1, 2], 30–70% of all ICU patients are, at one time, deeply sedated [3, 4]. Indeed, deep sedation, usually defined by a Richmond Assessment Sedation Scale (RASS) beneath −3, may be required in several conditions, including severe respiratory failure, septic shock, or controlling intracranial hypertension in severely brain-injured patients. However, this practice raises major concerns since the use of deep sedation has been incriminated in brain dysfunction [4, 5] and may contribute to increase the prevalence of delirium [6–8] and mortality [9, 10]. Indeed, more than half of all critically ill patients develop delirium, which is associated with a greater risk of death and long-term cognitive dysfunction [11–15]. Furthermore, severe brain injury may cause long-term disability or even be life-threatening [16]. Therefore, assessing and monitoring brain dysfunction in deeply sedated patient and determining its impact on outcome are major issues in the daily management of critically ill patients [17–21]. Neurophysiological testing enables assessment of brain dysfunction at the bedside [22]. Somatosensory evoked potentials (SSEP) and brainstem auditory evoked potentials (BAEP) explore the brainstem, as well as cortical and subcortical regions of the brain and are little influenced by the administration of sedatives [23–25]. More specifically, inter-peak latencies (IPL) of the components of the SSEP and BAEP are determined by conduction times between neuroanatomical regions or structures [26] and provide information about the functional state of the given brain portion [24, 27]. The prognostic values of SSEP and BAEP have been explored in various causes of coma but never in a cohort of deeply sedated critically ill patients [17, 19–21]. We hypothesized that the occurrence of brain dysfunction was associated with impaired intracranial (ICCT) and intrapontine (IPCT) conduction times assessed, respectively, by SSEP and BAEP. This bi-center prospective pilot cohort study was designed to determine whether early impairment of ICCT and IPCT could predict in-ICU mortality and the occurrence of post-sedation altered mental status (delirium or delayed awakening) in deeply sedated critically ill patients.

## Methods

### Study design and setting

This prospective observational study is reported following the Strengthening the Reporting of Observational Studies in Epidemiology (STROBE) guidelines [28]. The current pilot study was preparatory to the design of a larger prospective multicenter study assessing the prognostic value of brainstem dysfunction in sedated critically ill patients (ClinicalTrials.gov number: NCT02395861). Subjects were enrolled between January 2012 and January 2015. Participating centers were a medical (center 1) and a surgical (center 2) ICU in two French teaching hospitals. The former unit specializes in the management of medical critically ill patients, while the latter specializes in the management of subjects suffering from traumatic brain injuries.

### Characteristics of participants

Consecutive critically ill patients deeply sedated on day 3 following admission were enrolled into the study, irrespective of the existence or not of a primary brain injury and underwent electrophysiological explorations. Deep sedation was defined as a Richmond Assessment Sedation Scale (RASS) <−3 [29]. All included patients were mechanically ventilated. Post-cardiac arrest and moribund patients, patients in whom cerebral death was suspected or for whom withdrawal of life-sustaining therapies had been decided, and patients suffering from preexisting of acquired neuropathies were not included. Hypothermia may influence evoked potentials’ conduction times [30–33]. To avoid any confounding effect related to temperature, no evoked potential was recorded while body temperature was below 35 °C.

### Baseline clinical data collection

Demographic characteristics (i.e., age, sex) as well as body weight, date and time of ICU admission, category of admission (medical or surgical), co-morbidities, preexisting risk factors for delirium, main cause of critical illness and brain injury, and the date and cause of initiation of mechanical ventilation were collected. Baseline data collection was performed following a previously described method [34, 35].

### Sedation and analgesia

The decision to initiate deep sedation and the subsequent management thereof were overseen by the physicians in charge of the patient, following recent guidelines [1, 2]. Sedation was administered through a continuous infusion of midazolam and/or propofol, in combination with sufentanil. Total cumulative doses of administered drugs at the time of neurophysiological examination were collected. In both centers, the depth of sedation was monitored using the RASS [29], recorded every 4 h until awakening. Sedation was interrupted daily in center 1 (in which non-brain-injured patients were referred) and administrated as a titration aiming at obtaining the desired RASS in center 2 (in which severe traumatic brain-injured patients were cared for) [29]. The time of onset, the reason for administration, and duration of deep sedation, as well as the time of awakening, defined by the occurrence of spontaneous opening of the eyes with RASS >−1, were collected.

### Neurological examination

At the time of inclusion, the Glasgow Coma Scale (GCS), the Full Outline of Unresponsiveness (FOUR) score [36], and the Richmond Agitation-Sedation Scale (RASS) and the cough reflex were assessed. RASS is a simple and reliable tool that is intuitive, easy to use, and informs on both agitation and sedation. The RASS has been validated in mechanically ventilated and sedated patients [29, 37].

### Evoked potential (EP) assessment and analysis

SSEP after stimulation of the median nerve and BAEP were recorded at the bedside by an experimented neurophysiologist (EA) for all studied patients. EPs were recorded following the guidelines of the International Federation of Clinical Neurophysiology [24, 38]. A Natus France, Dantec™ KEYPOINT^®^ G4 EMG/NCS/EP Workstation was used for data acquisition and processing. All recordings were separately interpreted by two experienced neurophysiologists (EA and RMK). Any series of SSEP or of BAEP recording contaminated by noise was rejected, and recording was repeated following the administration of a neuromuscular blocking agent in order to eliminate muscular artifacts [24, 38].

#### Somatosensory evoked potentials (SSEP)

SSEP were recorded following stimulation of the median nerve at the wrist using repeated square-wave pulses lasting 0.2 ms, at 4.7 Hz, with stimulus intensity sufficient to produce a twitching of the thumb (motor threshold). Recording of the proximal peripheral nerve (brachial plexus) response N9 was performed through an electrode placed at the ipsilateral Erb’s point, while the reference electrode was situated over the centro-frontal region (Fz). Recording and reference electrodes were placed at Cv7 (7th cervical vertebra)—Fz for the N13 cervical spinal cord response and Cz—cSh (contralateral shoulder) for the subcortical far-field potential: P14. The cortical components, N20 and P25, were recorded at the contralateral C3′ or C4′ positions (2 cm behind C3 or C4) according to the international 10–20 system. Impedance was kept below 5 kOhms. The filter pass band ranged from 30 to 1500 Hz. Two sets of 500 sweeps were averaged. Figure 2—Appendix shows a schematic representation of median nerve’s SSEP responses localizations on a brain MRI (A) and typical examples of their normal waveforms as well as recording electrodes montages (B). Peak latencies (PL) of N9, N13, P14, and N20 responses, as well as IPL N9–N13 and P14–N20 IPLs, i.e., conduction times, were measured. N9–N13 IPL represents a proximal peripheral nerve conduction time, and P14–N20 IPL, the intracranial conduction time (ICCT). The absolute amplitude of the peak of N20 was measured. Each parameter was measured on both right and left median nerves for each patient. N20 component was considered absent (abolished) when its absolute amplitude did not exceed 0.1 µV.

#### Brainstem auditory evoked potentials (BAEP)

BAEP were recorded following auditory stimulation by a 100-µs 80-dB click applied to one ear, with a (−20 dB) contralateral masking using “white noise.” The recurrence frequency was 19.3 Hz (bandpass, 150–1500 Hz; sweep time, 10 ms). Two sets of 2000 sweeps were averaged. BAEP were picked up in Fz. BAEP recording in the Fz location of the electrode is more convenient when patients are supine since this location is easier to access than the vertex (Cz). The reference electrode was placed at the earlobe ipsilateral to the stimulated ear. Figure 3—Appendix shows a schematic representation of BAEP responses localizations on a brain MRI (A) and typical examples of their normal waveforms (B). PL of waves I, III, and V, as well as I–III, III–V, and I–V IPL, were measured. The I–III IPL represents the peripheral conduction time of the auditory pathway, while the III–V IPL represents the intrapontine conduction time (IPCT). Recordings of both right and left ears were made for all the patients.

### Evoked potentials data analysis

For each SSEP or BAEP parameter, the mean value of the left and right side was used for analysis. PL or IPL was considered delayed when they exceeded the mean value +2.5 SD obtained from a control group of 20 healthy subjects in our laboratory. Table 1 provides median nerve SSEP and BAEP’s PL and IPL data obtained our control group.

**Table 1:** Neurophysiological data

Evoked potentials data	Healthy control subjects (*n* = 20)		Deeply sedated critically ill patients (*n* = 86)	
	Mean latency ± SD (in ms)	Mean latency + 2.5 SD (in ms)	Mean latency ± SD (in ms)	Delayed latency *n* (%)
SSEP components				
N9 PL	9.8 ± 0.6	11.4	11.0 ± 1.6	33 (38)
N13 PL	13.2 ± 0.8	15.2	14.9 ± 2.1	36 (42)
P14 PL	14.6 ± 0.9	16.8	16.2 ± 2.0	32 (37)
N20 PL^a^	18.8 ± 1.0	21.4	21.6 ± 2.6	41 (48)
N9-N13 IPL	3.4 ± 0.4	4.4	3.9 ± 0.9	14 (16)
P14-N20 IPL^a^ (ICCT)	4.1 ± 0.5	5.3	5.3 ± 1.5	39 (45)
BAEP components				
Wave I PL	1.5 ± 0.2	2.0	1.69 (0.31)	13 (15)
Wave III PL	3.6 ± 0.2	4.1	3.96 (0.35)	34 (40)
Wave V PL	5.6 ± 0.3	6.3	6.16 (0.61)	34 (40)
I–III IPL	2.1 ± 0.2	2.6	2.28 (0.29)	17 (20)
III–V IPL (IPCT)	2.0 ± 0.2	2.5	2.20 (0.45)	15 (17)
I–V IPL	4.1 ± 0.3	4.8	4.51 (0.59)	17 (20)

*PL* peak latency, *IPL* inter-peak latency, *ICCT* intracranial conduction time, *IPCT* intra- pontine conduction time. PL and IPL of SSEP or BAEP’s components were scored as “delayed” when they were greater than the “mean + 2.5 SD” of the ones of a healthy control group

^a^N20 was abolished in three patients. N20 PL and P14-N20 IPL were consequently considered as delayed for these 3 patients with abolished N20. Median nerve somatosensory evoked potentials (SSEP) and brainstem auditory evoked potentials (BAEP) components latencies (PL) and inter-peak latencies (IPL) obtained from a group of 20 healthy control subjects (11 women, 9 men, mean age 51 ± 17 years) in our laboratory; and from 86 deeply sedated critically ill patients. Each variable is represented by the mean values of left and right hand sides

### Method for bias and confounding factors assessment

Confounding factors which may influence neurological examination, neurophysiological tests, mortality, and the occurrence of delirium were assessed. Neurological examination was performed by a specifically trained senior ICU physician. It has previously been shown that inter-observer agreement for such an examination was satisfactory (kappa scores ranged from 0.62 to 1) [35]. Neurophysiological tests were performed and interpreted in a standard manner following international guidelines [24, 38]. Each evoked potential recording was independently analyzed offline by two senior neurophysiologists (EA and RMK) who were blinded to clinical data. We assessed inter-observer rate of concordance for the studied evoked potential parameters using the kappa analysis. The physician in charge of the patient was not informed of the results of the neurophysiological test and the neurophysiologist was blinded to the clinical status and outcome of patients. Management of sedation was assessed by recording the indication of initiation, the daily RASS, modality of discontinuation (daily interruption *versus* titration) and duration. The cause of death and its main risk factors were also assessed, using the SAPS II and SOFA scores as well as the cause of critical illness. We were therefore able not only to compare subgroups but also to ensure that the management of sedation was appropriate and that the studied population was representative of French ICUs patients.

### Outcome assessment

The primary outcome was mortality in the ICU. We collected both the date and cause of death. Secondary outcome was the occurrence of altered mental status defined as either delirium or delayed awakening after discontinuation of sedation. Delayed awakening and the occurrence of delirium following discontinuation of sedation were assessed daily using the RASS and the Confusion Assessment Method in ICU (CAM-ICU), respectively [39]. Delayed awakening was defined by absence of spontaneous eye opening with RASS ≤−1 more than 3 days after discontinuation of sedation. Finally, the duration of mechanical ventilation and length of stay in the ICU were recorded.

### Statistical analysis

No published data enabled us to calculate a number of subject to be recruited, since the predictive values of SSEP and BAEP’s components IPL in deeply sedated critically ill patients have never been previously assessed. The present study was a pilot study for the design of a prospective multicenter study on the prognostic value of SSEP and BAEP in the ICU (ClinicalTrials.gov number: NCT02395861). We estimated that 80–100 included patients would be sufficient to test predictors of in-ICU mortality (primary objective). Data are reported as numbers (percentage), mean (standard deviation), or median (inter-quartile range). Groups were compared using the Mann–Whitney rank-sum test. Multivariable logistic regression was used to explore associations between impaired conduction times and in-ICU mortality adjusted to the global SOFA score and the non-neurological SOFA score. *p* values <0.05 were considered as statistically significant.

## Results

### Patients’ characteristics

**Fig. 1:**
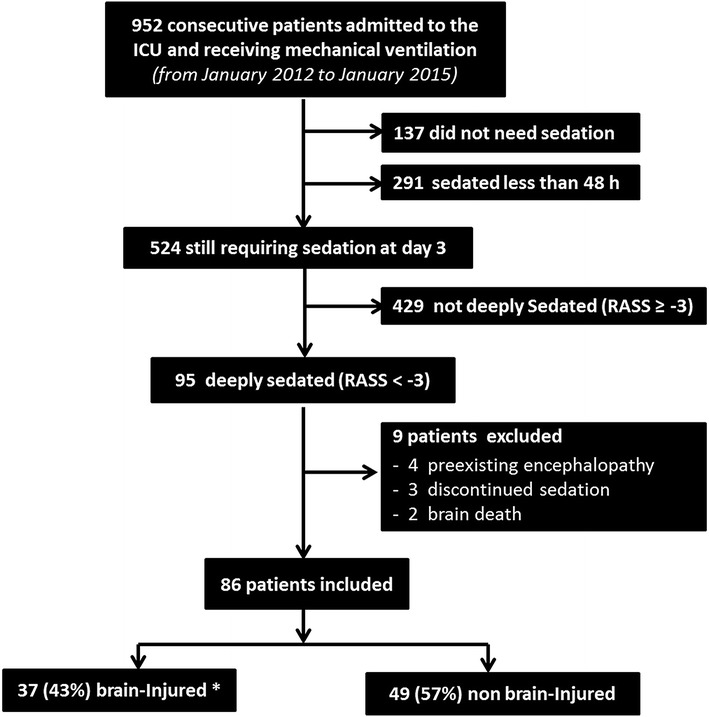
Flowchart. *Brain-injured patient was defined by admission to the ICU for acute brain injury

**Fig. 2:**
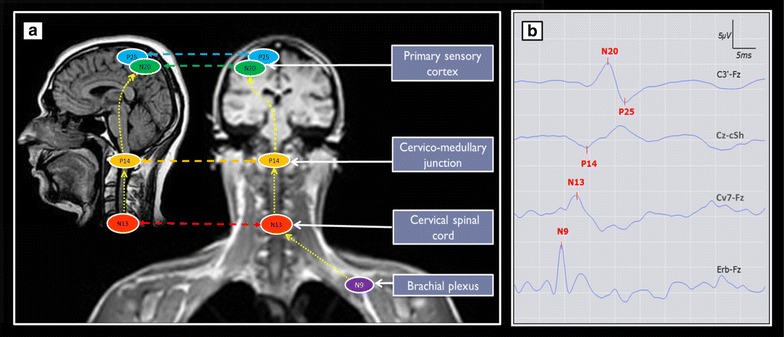
Schematic representation of median nerve’s somatosensory evoked potentials (SSEP) responses localizations on brain MRI (**a**) and typical examples of their normal wave forms as well as recording electrodes montages (**b**). SSEP elicited by electric stimulation (15 mA) of median nerve at the wrist: *N9*, *N13* and *P14*, respectively, the brachial plexus, cervical spinal cord, and cervico-medullary (subcortical) responses. *N20* and *P25* are responses of the primary sensory cortex. *N9*–*N13* inter-peak latencies (IPL) represent a proximal peripheral nerve conduction time, and *P14*–*N20* IPL the intracranial conduction time (ICCT). Recording and reference electrodes were placed at Cv7 (7th cervical vertebra)—Fz: for the *N13* cervical spinal cord response and Cz-cSh (contralateral shoulder): for the subcortical far-field potential: *P14*. The cortical components, *N20* and *P25* were recorded at the contralateral *C3′* or *C4′* positions (2 cm behind C3 or C4) according to the international 10–20 system. Two sets of 500 sweeps were averaged

**Fig. 3:**
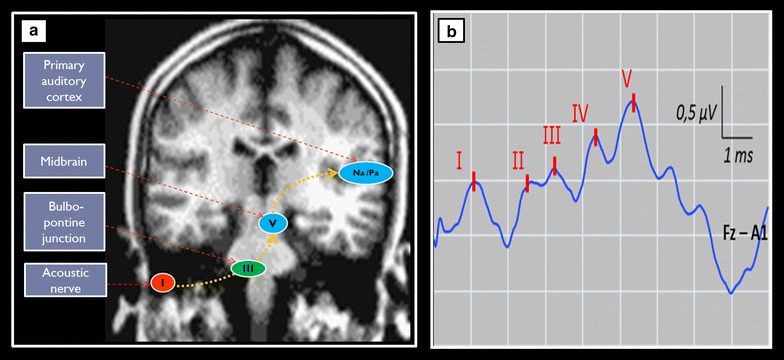
Schematic representation of brainstem auditory evoked potentials (BAEP) responses localizations on a brain MRI (**a**) and typical examples of their normal wave forms as well as recording montage (**b**). BAEP elicited by auditory click (100-µs 80-dB) applied to one ear, with a (−20 dB) masking of the contralateral ear by “white noise.” The recurrence frequency was 19.3 Hz (bandpass, 150–1500 Hz; sweep time, 10 ms). BAEP were picked up in Fz. with the reference electrode placed at the earlobe ipsilateral to the stimulated ear (Fz—A1 or A2). Two sets of 2000 sweeps were averaged. The *I*–*III* IPL represents the peripheral conduction time of the auditory pathway, when the *III*–*V* IPL represents the intrapontine conduction time (IPCT)

Between January 2012 and January 2015, 95 consecutive sedated critically ill patients were eligible for inclusion (Fig. 1). Nine patients were not included: Two patients exhibited clinical features of brain death, and for three patients, sedation had been discontinued prior to inclusion. In addition, we did not include four patients with severe encephalopathies related to either liver failure (2 cases) or status epilepticus (2 cases). Overall, 86 patients were enrolled; their baseline characteristics are presented in Table 2. Cause of ICU admission was neurological in 37 (43%) patients, mainly traumatic brain injury (TBI) 54%. Non-neurological critical illness related to sepsis was the cause of admission in 49 (57%) patients. Deep sedation was administrated at the time of inclusion for synchronization with the ventilator in 44 (51%) cases, for control of severe intracranial hypertension in 16 (19%) cases and for other vital causes in 26 (30%) cases.

**Table 2:** Patients’ characteristics

Variables	All patients	Non-brain-injured patients	Brain-injured patients	*p* value
Number of patients	86	49 (57)	37 (43)	
Women—*n* (%)	29 (34)	15 (31)	14 (38)	0.50
Age (years)—mean ± SD	61 ± 19	63 ± 16	58 ± 21	0.21
SAPS II at admission—median (IQR)	48 (36 to 61)	45 (30 to 61)	50 (41 to 58)	0.45
At the time of inclusion				
Sepsis—*n* (%)	56 (65)	46 (94)	10 (27)	<0.0001
RASS	−5 (−5 to −4)	−5 (−5 to −4)	−5 (−5 to −5)	0.82
Use of sedation—*n* (%)				<0.0001
Synchronization with ventilator	44 (51)	33(67)	11 (30)	
Intracranial hypertension	16 (19)	0 (0)	16 (43)	
Others	26 (30)	16 (33)	10 (27)	
Sedative drug used				
Midazolam—*n* (%)	79 (92)	48 (98)	31 (84)	0.02
Sufentanyl—*n* (%)	79 (92)	47 (96)	32 (86)	0.40
Propofol—*n* (%)	9 (10)	3 (6)	6 (16)	0.13
Total day-3 SOFA—median (IQR)	11 (8 to 14)	13 (10 to 15)	10 (8 to 11)	0.0006
Non-neurological day-3 SOFA—median (IQR)	7 (5 to 10)	9 (6 to 11)	6 (4 to 7)	0.052
Renal day-3 SOFA—median (IQR)	0 (0 to 2)	1 (0 to 3)	0 (0 to 1)	0.03
Liver day-3 SOFA—median (IQR)	0 (0 to 1)	0 (0 to 1)	0 (0 to 0)	0.19
Outcomes				
Duration of sedation (days)—median (IQR)	7 (4 to 14)	7 (5 to 14)	8 (4 to 12)	0.48
Duration of mechanical ventilation (days)—median (IQR)	15 (7 to 30)	10 (6 to 34)	18 (10 to 27)	0.15
Delirium—*n* (%)	35/61 (57)	17/30 (57)	18/31 (58)	>0.99
Delayed awakening—*n* (%)	23/63 (37)	7/32 (22)	16/31 (52)	0.02
Altered mental status—*n* (%)	49/63 (78)	21/31 (68)	28/32 (88)	0.07
In-ICU mortality—*n* (%)	37/86 (43)	24/49 (49)	13/37 (35)	0.19

*SAPS II* Simplified Acute Physiology Score, *EP* evoked potentials, *EEG* electroencephalogram, *SIRS* Systemic Inflammatory Response Syndrome, *RASS* Richmond Assessment Sedation Scale, *SOFA* Sepsis-related organ failure assessment, *ICU* intensive care unit, delirium was defined according to the CAM-ICU

### Clinical and neurophysiological characteristics of the patients at inclusion

At the time of inclusion (i.e., time of EP recording), the median RASS was −5 [−5 to −4] (Table 2). Temperature values ranged from 35.1 to 39.3 °C with a mean (±SD) of 36.6 (±1.0) °C. Administered hypnotics were midazolam in 79 (92%) cases and propofol in 9 (10%) cases; 2 (2%) patients received both drugs simultaneously. Sufentanil was administered to 79 (92%) patients, while neuromuscular blocking agents were administered to 13 (15%) patients. Main neurological and neurophysiological features are presented in Table 3. Recordings from all included patients were deemed suitable for analysis. Inter-observer agreement for interpreting EP findings was excellent. Observed agreement was 100% (kappa = 1) for peak detection and placement of markers. The prevalence of delayed IPL of SSEP’s components increased from the periphery to the cortical level: proximal peripheral nerve conduction time (i.e., N9–N13 IPL) was prolonged in 14 (16%) of cases whereas the intracranial conduction time—ICCT (i.e., P14–N20 IPL) was prolonged in 39 (45%) of cases. I–III and I–V IPL were prolonged in 17 (20%) and the intrapontine conduction time—IPCT (i.e., III–V IPL) in 15 (17%) of cases (Tables 1, 3). SSEP N20 was unilaterally abolished in 3 patients. P14–N20 IPL was consequently considered as delayed for these 3 patients with abolished N20. No case with bilaterally abolished N20 was found. Brain imaging was performed in 59 (69%) patients using conventional CT or MRI in, respectively, 36 (61%) and 23 (39%) patients. Brain imaging was normal in 25 (42%) patients. Among the 34 (58%) patients with abnormal imaging, no brainstem injury was reported.

**Table 3:** Patient’s neurological and neurophysiological characteristics at time of inclusion

Variables	All patients	Non-brain-injured patients	Brain-injured patients	*p* value
Number of patients	86	49 (57)	37 (43)	
Glasgow Coma Score—median (IQR)	3 (3 to 3)	3 (3 to 3)	3 (3 to 3)	0.97
FOUR score—median (IQR)	4 (2 to 5.7)	4 (2 to 5.0)	4 (2 to 5.5)	0.73
Abolition of cough reflex	24 (29)	16 (33)	8 (26)	0.34
RASS—median (IQR)	−5 (−5 to −4)	−5 (−5 to −4)	−5 (−5 to −5)	0.82
Delayed SSEP’s IPL—n (%)				
N9–N13 IPL	14 (16)	11 (22)	3 (8)	0.08
P14–N20 IPL^a^ (ICCT)	39 (45)	21 (43)	18 (49)	0.60
Delayed BAEP’s IPL—*n* (%)				
I–III IPL	17 (20)	9 (18)	8 (22)	0.71
III–V IPL (IPCT)	15 (17)	9 (18)	6 (16)	0.80
I–V IPL	17 (20)	12 (24)	5 (13)	0.21

*FOUR* the Full Outline of Unresponsiveness (FOUR) score, *RASS* Richmond Assessment Sedation Scale, *PL* peak latency, *IPL* inter-peak latency, *ICCT* intracranial conduction time, *IPCT* intra pontine conduction time. PL and IPL of SSEP or BAEP’s components were scored as “delayed” when they were greater than the “mean + 2.5 SD” of the ones of a healthy control group

^a^N20 was abolished in three patients. N20 PL and P14–N20 IPL were consequently considered as delayed for these three patients

### Comparisons of clinical and neurophysiological findings between the two subgroups of patients: brain-injured versus non-brain-injured group

Prevalence of sepsis, day 3 global SOFA, renal SOFA scores, and proportion of patients sedated with midazolam were significantly greater in non-brain-injured patients (Table 2). The median GCS, the FOUR scores, and the median RASS were not significantly different between the two groups (Table 3). The two groups did not differ in terms of abolition of cough reflex and conduction times (Table 3). Prevalence of delayed conduction times did not differ between subgroups of patients with and without abnormal brain imaging. Conduction times were neither statistically correlated with cumulative doses of midazolam, propofol, or sufentanil at inclusion, and no significant difference exists between temperature values of patients with versus without delayed conduction times (Table 5).

### Correlations between clinical, neurophysiological features, and in-ICU mortality

Thirty-seven patients (43%) died in the ICU, of which 24 (65%) were non-brain-injured patients. Causes of death were refractory hypotension in 28 (76%) patients, respiratory failure in five cases (13%), and brain death in four (11%) patients. Most non-brain-injured patients died of refractory hypotension 18 (75%), and most brain-injured patients died of refractory intracranial hypertension 4 (31%). Withdrawal of life-sustaining therapies was decided in 6 (7%) patients and was never decided based on the results of evoked potential recordings. Comparisons of clinical and neurophysiological features between survivors and non-survivors appear in Table 4. The SOFA score was significantly higher in non-survivor (*p* = 0.004). The RASS and FOUR scores were significantly lower in non-survivors (*p* = 0.001 and *p* = 0.026, respectively). Impaired ICCT (i.e., delayed SSEP’s P14–N20 IPL) and abolition of cough reflex, were significantly associated to in-ICU mortality (*p* = 0.029, *p* = 0.003, respectively). On multivariate analysis, impaired ICCT was independently associated with in-ICU mortality after adjustment to the total SOFA score [OR (95% CI) = 2.69 (1.05–6.85) *p* = 0.039] and after adjustment to non-neurological SOFA score [OR (95% CI) = 2.67 (1.05–6.81) *p* = 0.040].

**Table 4:** Comparison of clinical and neurophysiological abnormalities among outcome categories

Variables	Survivors	Non-survivors	*p* value	No altered mental status	Altered mental status	*p* value
Number of patients—*n* (%)	49 (57)	37 (43)		14 (22)	49 (78)	
Age (years)—mean (SD)	58 (18)	64 (19)	0.058	58 (20)	60 (20)	0.75
Women—*n* (%)	19 (39)	10 (27)	0.36	5 (36)	20 (41)	>0.99
SAPS II—median (IQR)	48 (37 to 60)	53 (34 to 70)	0.47	45 (28 to 53)	48 (37 to 59)	0.36
Day-3 SOFA—median (IQR)	10 (8 to 12)	13 (10 to 17)	0.004	10 (8 to 12)	10 (8 to 13)	0.96
GCS—median (IQR)	3 (3 to 4)	3 (3 to 3)	0.18	3 (3 to 3)	3 (3 to 5)	0.29
FOUR Score—median (IQR)	4 (3 to 6.5)	4 (0 to 4.0)	0.026	4 (3.2 to 5)	4 (3 to 7)	0.85
Abolition of cough reflex-*n* (%)	7 (15)	17 (47)	0.003	3 (21)	10 (22)	>0.99
RASS^a^—median (IQR)	−5 (−5 to −4)	−5 (−5 to −5)	0.001	−5 (−5 to −4)	−5 (−5 to −4)	0.95
Brain injured—*n* (%)	24 (49)	13 (35)	0.27	4 (29)	28 (57)	0.07
Sedative drug used						
Midazolam—*n* (%)	44 (90)	35 (95)	0.43	13 (93)	44 (90)	>0.99
Sufentanil—*n* (%)	44 (90)	35 (95)	0.86	13 (93)	44 (90)	>0.99
Propofol—*n* (%)	9 (18)	0 (0)	0.009	0 (0)	8 (16)	0.18
Conduction times (ms)—mean (SD)						
SSEP N9–N13 IPL	3.8 (0.9)	4 (0.9)	0.17	3.7 (0.6)	3.9 (0.9)	>0.99
SSEP P14–N20 IPL (ICCT)^a^	5.2 (1.6)	5.6 (1.5)	0.02	5.1 (1.3)	5.4 (1.6)	0.64
BAEP I–III IPL	2.2 (0.3)	2.4 (0.3)	0.09	2.3 (0.3)	2.3 (0.3)	0.81
BAEP III–V IPL (IPCT)	2.2 (0.5)	2.3 (0.4)	0.33	2.0 (0.3)	2.3 (0.5)	0.001
Delayed conduction time—*n* (%)						
SSEP N9–N13 IPL	7 (14)	7 (19)	0.57	1 (7)	6 (12)	>0.99
SSEP P14–N20 IPL	17 (35)	22 (59)	0.029	5 (36)	22 (45)	0.76
BAEP I–III IPL	9 (18)	8 (22)	0.79	3 (21)	7 (14)	0.68
BAEP III–V IPL	8 (16)	7 (19)	0.78	0 (0)	12 (24)	0.053

*IPL* inter-peak latency. *ICCT* intracranial conduction time, *IPCT* intra- pontine conduction time; IPL of SSEP or BAEP’s components were scored as “delayed” when they were greater than the “mean + 2.5 SD” of the ones of a healthy control group

^a^N20 was abolished in three patients. P14–N20 IPL were consequently considered as delayed for these three patients with abolished N20

### Correlations between clinical, neurophysiological features, and the occurrence of an altered mental status (AMS)

Median duration of sedation after inclusion was 7 days [4–14]. Twenty-three (27%) patients died before discontinuation of sedation. Among the 63 (73%) remaining patients, 49 (78%) patients developed an altered mental status, with delirium occurring in 35 (57%) cases and delayed awakening in 23 (63%) cases. Delayed awakening, but not delirium was significantly more frequent among brain-injured patients (*p* = 0.02) (Table 4). Median duration of delirium was 5 days [3–17]. No patient evolved toward a vegetative or minimally conscious state. There was no difference between patients with and without altered mental status in terms of demography, cause and severity of critical illness, brainstem reflexes or sedation (Table 4). Impaired IPCT (i.e., delayed BAEP’s III-V IPL) was more frequent among the subgroup of patients who developed AMS compared with the one without AMS (24 vs. 0%); however, this association did not reach statistical significance (*p* = 0.053).

## Discussion

In the present study, we found that in critically ill patients receiving deep sedation, early impairment of ICCT (i.e., delayed SSEP’s P14–N20 IPL) adjusted to patient severity (day 3 SOFA score), predicted in-ICU mortality, and that impairment of IPCT (i.e., delayed III–V IPL) tended to be associated with the occurrence of an altered mental status. We also found an impaired conduction time at the peripheral level, suggesting that neurological dysfunction of these patients affects both the central and the peripheral compartments. These findings support the fact that ICCT and IPCT are useful early warning indicators of brain dysfunction and prognosis markers in deeply sedated critically ill patients in ICU.

Neurophysiological assessment of comatose critically ill patients using evoked potentials have multiple advantages; being noninvasive, available at the bedside, capable of detecting subclinical injuries or providing objective measures when information derived from the clinical examination is poor [22]. Evoked potentials have already been extensively studied when seeking to determine a prognosis following coma from various origins [18, 19, 26, 40–44]; however, little is known regarding critically ill patients receiving deep sedation, which may be required in severe critically ill patients who are at high risk of developing acute brain dysfunction.

Sedative drugs, at least those used during our study, mainly act on cortical receptors [24, 45]. Deep sedation has been reported to be associated with increased ICU mortality [9, 10, 37, 46]. Critically, short-latency evoked potentials used in this study are largely unaffected by commonly used sedative drugs [24, 45]. Indeed, we did not find a correlation between conduction times and sedation doses. To ensure that deep sedation was not involved in the delay of ICCT and IPCT, it would have been necessary to modify the infusion rate of sedative agents. However, such an intervention was prohibited as our study was strictly observational and since sedation was managed by the physician in charge of the patient. The observed delay of intracranial conduction time therefore probably resulted from subcortical demyelinating [47, 48] and/or axonal insults [49, 50]. These lesions have already been described in neuropathological or neuroradiological studies of various acute brain dysfunctions, notably traumatic brain injury [16, 17] and critical illness [51–53].

Our findings provide valuable information on the incidence of secondary brain insults occurring in the ICU, in both primary and non-primary brain-injured patients. While delayed intracranial conduction time was expected in the group of brain-injured patients, it was found with a similar incidence in non-primary brain-injured patients. This finding suggests that secondary insults occur in both primary brain-injured and non-brain-injured patients, homogenizing these two subgroups in terms of central conduction times. Both primary brain-injured and non-brain-injured patients are liable to secondary insults, including ischemic, metabolic, toxic or inflammatory factors [54]. Therefore, this finding would also indicate that medical critically ill patients should potentially be considered brain injured. Brain injury and insults have been documented in septic patients. Therefore, one may argue that the dichotomy between brain injured and non-brain injured is inaccurate and that a dichotomy between primary versus secondary brain insult should be preferred (Table 5).

**Table 5:** Comparing sedative drug use and cumulative administered dose among patients with and without delayed intracranial conduction time

Variables	All patients	SSEP’s P14–N20 (ICCT)			BAEP’s III–V (IPCT)		
		Delayed	Non delayed	*p value	Delayed	Non delayed	*p value
Number of patients—*n* (%)	86 (100)	39 (45)	47 (55)		15 (17)	71 (83)	
RASS—median (IQR)	−5 (−5 to −4.2)	−5 (−5 to −5)	−5 (−5 to −4)	0.003	−5 (−5 to −4)	−5 (−5 to −5)	0.45
Body temperature (°C)—mean (SD)	36. (1.0)	36.7 (1.0)	36.4 (0.9)	0.10	36.5 (1.1)	36.6 (1.0)	0.67
Sedative drugs used							
Midazolam—*n* (%)	79 (92)	36 (42)	43 (50)	0.89	14 (18)	65 (82)	0.82
Sufentanil—*n* (%)	79 (92)	37 (43)	42 (49)	0.36	15 (19)	64 (81)	0.21
Propofol—*n* (%)	9 (10)	2 (2)	7 (8)	0.14	1 (11)	8 (89)	0.60
Sedative drugs’ cumulative doses							
Midazolam (mg/kg)—median (IQR)	5 (5 to 8)	5 (5 to 9)	5 (5 to 8)	0.74	5 (4.2 to 9)	5 (5 to 8)	0.54
Sufentanil (µg/kg)—median (IQR)	20 (10 to 30)	20 (10 to 30)	20 (10 to 30)	0.70	20 (10 to 25)	20 (10 to 30)	0.63
Propofol (mg/kg)—median (IQR)	10 (0 to 40)	3 (1.5 to 6)	20 (1.5 to 85)	0.22	3 (3 to 3)	15 (0 to 55)	0.40
Abnormal brain imaging—*n* (%)	34/59 (58)	18/34 (53)	16/34 (47)	0.08	5/34 (15)	29/34 (85)	0.46
Focal brain injury—*n* (%)	11 (32)	6 (55)	5 (45)	0.96	1 (9)	10 (91)	0.72
Diffuse brain injury—*n* (%)	23 (68)	12 (52)	11 (48)	0.78	4 (17)	19 (83)	0.66

* Mann–Whitney test

While delayed intracranial conduction time is probably caused by secondary brain insults and to a lesser extent, by sedatives, its association with mortality may be explained by the extent of cerebral suffering, including involvement of critical areas implicated in preserving vital functions such as the brain stem [34, 35, 55]. Delirium, characterized by impaired cognition, conscience and arousal, is frequent in the ICU [6–8]. Impairment of the ascending reticular activation system (ARAS) located in the upper part of the brainstem may also be implicated in the genesis of delirium, notably impairment of arousal. Interestingly, increased IPCT may reflect a dysfunction at the level of pons or midbrain, which includes the ARAS [56]. Finally, impaired intracranial conduction times have long been evidenced in various primary brain insults; that are also associated with delirium [26, 44].

## Study limitations

Our study has several limitations. First, since our aim was to assess the usefulness of evoked potential in patients requiring deep sedation, we studied both primarily brain-injured and non-brain-injured patients. Identifying neurophysiological differences between brain-injured and non-brain-injured patients and between septic and non-septic patients might be hampered by a lack of power. Due to the observational nature of the study, we are unable to determine whether the observed impaired consciousness results from brain insult, sedation or both. Finally, since we did not adjust statistical tests for multiple comparisons, our results should be viewed as exploratory. This limitation is mitigated by the fact that we tested a small number of scientific hypotheses—those pertaining to the association of EPs with the outcomes.

Overall, in deeply sedated critically ill patients, early impairment of ICCT was associated with in-ICU mortality while early impairment of IPCT tended to be associated with the occurrence of altered mental status. Confirmation of these results is currently under investigation in a larger multicenter prospective cohort study (ClinicalTrials.gov number: NCT02395861).

## Appendix

See Figs. 2 and 3.

## Abbreviations

AMS: altered mental status
BAEP: brainstem auditory evoked potentials
CAM-ICU: Confusion Assessment Method in ICU
EEG: electroencephalogram
EP: evoked potential
FOUR: the Full Outline of Unresponsiveness score
GCS: the Glasgow Coma Scale
ICCT: intracranial conduction time
ICU: intensive care unit
IPCT: intrapontine conduction time
IPL: inter-peak latency
MCA: multiple correspondence analysis
PL: peak latency
RASS: Richmond Assessment Sedation Scale
SAPS II: Simplified Acute Physiology Score
SIRS: Systemic Inflammatory Response Syndrome
SOFA: Sequential Organ Failure Assessment score
SSEP: somatosensory evoked potentials
STROBE: Strengthening the Reporting of Observational Studies in Epidemiology
TBI: traumatic brain injury
